# Fermentation and Refrigerated Storage of Millet-Based Beverages with Yoghurt and Propionic Acid Bacteria

**DOI:** 10.3390/microorganisms14102288

**Published:** 2026-10-08

**Authors:** Małgorzata Ziarno, Dorota Zaręba, Rozeta Hasalliu

**Affiliations:** 1 Department of Food Technology and Assessment, Faculty of Bioeconomy and Food Systems, Warsaw University of Life Sciences—SGGW (WULS–SGGW), Nowoursynowska 159c St., 02-776 Warsaw, Poland; 2 Professor E. Pijanowski Catering School Complex in Warsaw, 04-110 Warsaw, Poland; dorotazareba@gmail.com; 3 Department of Food Science and Biotechnology, Faculty of Food Biotechnology, Agricultural University of Tirana, Rruga “Pajsi Vodica”, Kodër-Kamëz, 1029 Tirana, Albania; rhasalliu@ubt.edu.al

**Keywords:** millet beverage, plant-based fermentation, yoghurt starter culture, *Propionibacterium freudenreichii*, co-fermentation, microbial viability, syneresis, refrigerated storage

## Abstract

Millet beverages require fermentation processes that provide acidification, culturable starter populations and resistance to serum separation. Beverages containing 0 to 3% (*w*/*v*) millet flour were fermented with a yoghurt starter, *Propionibacterium freudenreichii* subsp. *shermanii*, or both cultures. Fermentation was conducted at 37 °C for 6 h with yoghurt or mixed cultures and 18 h with the propionic starter, followed by storage at 6 °C for 28 days. Two independent production experiments were performed, each with duplicate samples. Acidification, bacterial counts, pH, penetration forces, centrifugal syneresis and selected carbohydrates were assessed. Mixed cultures yielded higher presumptive propionibacteria counts after fermentation and storage, reaching 6.7 log CFU/mL on day 28 with 2% flour. Their pH remained approximately 4.1–4.2. Flour increased hardness and reduced centrifugal serum release. With 3% flour, syneresis on day 28 was 48.3% for yoghurt and 57.8% for mixed cultures. Carbohydrate concentrations below 0.50 mg/g were treated as semiquantitative estimates. The results describe formulation-dependent technological responses without establishing metabolic synergy. Limited independent replication, substantial centrifugal serum release and the absence of rheological and sensory measurements restrict statistical inference and conclusions about commercial suitability.

## 1. Introduction

Plant-based beverages have moved from a niche category to a widely studied group of dairy alternatives. Their development is driven by dietary diversification, lactose intolerance, milk protein allergy, environmental concerns, and consumer interest in plant-based foods. However, replacing milk with a cereal suspension is not a simple substitution. Cereal beverages usually contain less protein, have a different amino acid profile, and lack the casein network that supports the texture of fermented milk. They are also prone to sedimentation, serum separation, and changes in viscosity during storage. Fermentation can improve flavour, microbial safety, digestibility, and the availability of selected nutrients, but its outcome depends on the compatibility between the plant matrix and the microorganisms used [1,2]. A starter culture that performs well in milk may acidify a cereal substrate slowly, lose viability, or produce a structure that is unsuitable for a beverage.

Millet is a promising raw material for fermented beverages because it is naturally gluten-free, tolerates drought, and can be cultivated under conditions in which many major cereals perform poorly. Millet grain supplies starch, dietary fibre, minerals, and phenolic compounds, although its composition varies substantially among species and cultivars [3]. Its wider use is limited by factors that also affect other whole grains, including phytate, tannins, low protein digestibility, and an amino acid pattern that does not fully meet human requirements. Processing and fermentation may reduce some antinutritional compounds and improve nutrient accessibility, while also generating acids and volatile metabolites that modify flavour [3,4]. The technological response of millet is nevertheless difficult to predict because starch, proteins, fibre, and suspended particles contribute simultaneously to buffering, water binding, viscosity, and phase stability.

Most studies on fermented millet foods have focused on traditional porridges, sour products, or beverages inoculated with lactic acid bacteria. Controlled fermentation has shown that millet can support high populations of selected lactobacilli and other starter organisms [4]. More recent work has demonstrated the feasibility of producing yoghurt-like finger millet beverages with probiotic and prebiotic components, but the final acidity, texture, and sensory properties depended strongly on formulation and culture selection [5]. Similar observations have been reported for fermented oat drinks, in which starter composition determined acidification kinetics, microbial succession, aroma development, and consumer perception [6]. These findings indicate that the value of fermentation cannot be assessed only from the final pH. Viable cell retention, post-acidification, carbohydrate conversion, water-holding capacity, and physical stability during refrigeration are equally relevant to product quality.

The conventional yoghurt consortium consists of *Streptococcus thermophilus* and *Lactobacillus delbrueckii* subsp. *bulgaricus*. In milk, these organisms exhibit protocooperation rather than simple parallel growth. Their interaction includes the exchange of peptides and amino acids, carbon dioxide, purine precursors, and other metabolites that accelerate growth and lactic acid formation [7]. The relationship is strain-dependent. Differences in proteolytic activity, metabolite exchange, and stress tolerance can alter the growth balance and the metabolite profile of the fermented product [8]. Transfer of this consortium from milk to a low-protein cereal matrix, therefore, raises two opposing expectations. The established metabolic cooperation may permit rapid acidification despite the absence of lactose and casein, but nutrient limitation and differences in buffering capacity may weaken the association or reduce survival during storage. Both outcomes have been reported in plant substrates, which makes direct experimental evaluation necessary.

*Propionibacterium freudenreichii* is traditionally associated with Swiss-type cheeses, where it converts lactate mainly into propionate, acetate, and carbon dioxide. Selected strains can also synthesise vitamin B12 and have attracted interest as food-grade cultures with functional potential [9]. Its behaviour in plant beverages is less predictable than in dairy systems. *P. freudenreichii* generally grows more slowly than thermophilic yoghurt bacteria, depends on accessible carbon and nitrogen sources, and responds strongly to temperature, redox conditions, and prior adaptation. A strain that performs poorly in a soy beverage as a monoculture may grow when combined with a lactic acid bacterium, which supports the hypothesis that lactate production and metabolite exchange can facilitate propionic fermentation [10]. Cooperative cultures have also enhanced vitamin B12 formation and carbohydrate conversion in sunflower seed beverages [11]. Conversely, the accumulation of organic acids, competition for nutrients, or an unsuitable incubation temperature may suppress one or more partners. Thus, the coexistence of yoghurt bacteria and *P. freudenreichii* should not be assumed to be synergistic in every plant matrix.

Evidence from plant beverages remains limited and matrix-specific. Propionibacteria have been maintained in fermented soy, almond, and coconut beverages, although survival during refrigeration depended on the accompanying probiotic starter and the substrate [12]. In rice-based beverages, combinations of lactic acid bacteria and Propionibacterium affected both acidification and the product’s physicochemical properties [13]. Studies on oat drinks further showed that fermentation by lactic acid bacteria, bifidobacteria, and propionibacteria can modify texture and water-holding capacity, but the effects are not interchangeable among cultures [14]. Taken together, these reports suggest that mixed fermentation may combine rapid acidification with improved propionibacteria persistence. They also show that microbiological performance and physical stability may require different formulations. This distinction is especially relevant for millet beverages, where a low native protein content limits gel formation, and the addition of flour can simultaneously increase total solids, starch, fibre, and suspended particles.

The addition of millet flour may therefore influence fermentation through several mechanisms. It can supply fermentable carbohydrates and growth factors, increase buffering capacity, and provide surfaces for microbial attachment. At the same time, gelatinised starch and hydrated fibre can increase viscosity and reduce serum release. A higher solids content is not necessarily advantageous for all responses. It may improve hardness and water retention while restricting nutrient diffusion, changing substrate accessibility, or intensifying competition between cultures. The resulting product must be evaluated as a biological and physical system, rather than as an acidified suspension alone. Information is still lacking on how moderate flour supplementation interacts with yoghurt bacteria and *P. freudenreichii* during both fermentation and refrigerated storage.

Traditional cereal fermentations provide a useful basis for developing plant-based dairy alternatives, but their reported nutritional effects cannot be assumed to apply to a new formulation without direct measurement. Horlacher et al. [15] emphasised the relationship between fermentation conditions, microbial communities and changes in plant nutrients. For millet beverages, a practical initial question is whether a defined starter combination can acidify the substrate, remain culturable during refrigeration, and limit serum release. Demonstrating these outcomes is distinct from demonstrating cross-feeding, nutritional improvement or a probiotic effect.

The aim of this study was to determine how millet flour supplementation and starter composition influence fermentation performance and the storage characteristics of millet-based beverages. The specific objectives were to compare pH development during fermentation, track culturable bacterial populations and pH over 28 days at 6 °C, evaluate hardness, adhesive force, and centrifugal syneresis, and quantify selected residual carbohydrates. The working hypothesis was that combining the yoghurt starter with *P. freudenreichii* would be associated with higher recoverable propionibacteria populations, whereas flour supplementation would primarily influence physical properties. The study was designed as a technological comparison under a common incubation temperature, rather than as a direct test of metabolic synergy.

## 2. Materials and Methods

### 2.1. Materials

A commercial millet beverage (Natumi GmbH, Troisdorf, Germany) and commercial millet flour (Radix-Bis Sp. z o.o., Rotmanka, Poland) were used to prepare the fermentation substrates. The millet beverage originated in the European Union. According to the nutritional declaration provided on the product label, 100 mL of the beverage supplied 226 kJ (54 kcal) and contained 1.1 g of fat, including 0.1 g of saturated fatty acids, 10.0 g of carbohydrates, including 5.5 g of sugars, less than 0.5 g of dietary fibre, 0.7 g of protein, and 0.08 g of salt.

The millet flour was produced in Poland. According to the nutritional declaration provided on the product label, 100 g of flour supplied 1449 kJ (346 kcal) and contained 2.9 g of fat, including 0.6 g of saturated fatty acids, 71.6 g of carbohydrates, including 1.0 g of sugars, 3.2 g of dietary fibre, 10.5 g of protein, and 0.01 g of salt. The nutritional composition of both raw materials was based on the manufacturers’ declarations and was not determined analytically in the present study.

Two commercial starter cultures for dairy applications were obtained from Dalton Biotecnologie S.r.l. (Spoltore, Italy). According to the manufacturer, POB 010 contained *Streptococcus thermophilus* and *Lactobacillus delbrueckii* subsp. *bulgaricus* and was characterised by rapid acidification and exopolysaccharide-producing capacity. Its recommended application temperature for yoghurt was 38–43 °C. PROPIONICI contained *Propionibacterium freudenreichii* subsp. *shermanii* and was intended for Swiss-type cheese manufacture and ripening. The recommended application temperature was 25 °C, with 15 to 20 °C also indicated as suitable. These are manufacturer specifications, not experimentally determined optima in millet. Strain identifiers were not available. Both lyophilised cultures were stored at −18 °C until use. EPS production in the millet beverages was not measured.

### 2.2. Beverage Preparation and Experimental Design

For each formulation, 600 mL of commercial millet beverage was supplemented with 0, 6, 12, or 18 g of millet flour, corresponding to 0, 1, 2, or 3% (*w*/*v*), respectively. The formulation without added flour served as the formulation control within each starter treatment. It was fermented and should not be interpreted as an uninoculated control.

The suspensions were mixed, heated at 85 °C for 10 min and cooled to 45 °C before inoculation. Yoghurt-only and propionic-only treatments received 0.04% (*w*/*w*) of their respective commercial starters. Mixed treatments received 0.04% (*w*/*w*) of each starter, giving 0.08% (*w*/*w*) total starter. The inoculated beverages were distributed into sterile 180 mL glass jars. All treatments were incubated at 37 °C. Fermentation lasted 6 h for yoghurt and mixed cultures and 18 h for propionibacteria alone. The same durations applied to the acidification profiles. After fermentation, samples were refrigerated at 6 °C for 28 days. Day 0 denotes the post-fermentation sampling point after cooling. Immediately after inoculation and before fermentation, bacterial counts were on the order of 10^7^ CFU/g of beverage in both single-starter and mixed treatments. This value describes the inoculated beverage, not the concentrated starter preparation. It is an approximate inoculum level and does not establish identical initial counts for each bacterial group or an equal total cell dose across treatments.

The common incubation temperature enabled comparison of the starter treatments under a single thermal condition. It was not an optimisation of temperature for either commercial culture. In particular, 37 °C exceeded the recommended application temperature for PROPIONICI. The different fermentation durations and the higher total starter dose in mixed treatments are relevant to the interpretation of the comparisons.

Two independent production experiments were performed using independent batches of raw materials. Each experiment included two replicate samples of every treatment. Separate jars were opened at successive sampling times. Thus, storage measurements did not involve repeated opening of the same jar. The independent production replication was *n* = 2; the duplicate samples within each experiment provided four sample observations per experimental combination but did not constitute four independent production batches.

### 2.3. Acidification Profiles

Beverage pH was monitored during incubation at 37 °C, from inoculation to 6 h for yoghurt and mixed cultures and to 18 h for the propionic starter. Measurements were taken at 1 h intervals using a CP-505 pH meter (Elmetron, Zabrze, Poland) with temperature compensation. Determinations were performed in duplicate and recorded to 0.01 pH units. Fermentation profiles were expressed as pH against time. These measurements describe net acidification and do not quantify organic acid production or metabolic flux.

### 2.4. Enumeration of Culturable Bacteria

Bacterial populations were enumerated on days 0, 14 and 28. Tenfold serial dilutions were prepared in sterile Ringer’s solution (Sigma-Aldrich, St. Louis, MO, USA), prepared according to the supplier’s instructions and autoclaved at 121 °C for 20 min. The dilution procedure used the general approach described in ISO 6887-5 [16], adapted to the beverage matrix. That standard concerns milk and milk products; it does not validate selective enumeration in cereal beverages. Culture media and incubation conditions were as previously described for fermented oat drinks [14].

Presumptive lactobacilli were enumerated on MRS agar (Merck, Darmstadt, Germany) incubated anaerobically at 37 °C for 72 h using Anaerocult jars (Merck, Darmstadt, Germany). Presumptive *S. thermophilus* was enumerated on M17 agar (Merck, Darmstadt, Germany) incubated aerobically at 37 °C for 72 h. For presumptive *Propionibacterium* spp., sodium lactate agar contained yeast extract, 10 g/L; sodium hydrogen phosphate monohydrate, 3 g/L; potassium dihydrogen phosphate, 1 g/L; sodium lactate, 40 g/L; and agar, 15 g/L [14]. The medium pH was adjusted to 7.0 to 7.2 at 25 °C, and plates were incubated anaerobically at 37 °C for 7 days.

The yoghurt starter alone was tested on the sodium lactate medium under the enumeration conditions used in this study, and no growth was observed. This control supports differentiation of the tested commercial starter combination under those conditions. It does not establish universal selectivity of sodium lactate agar for *P. freudenreichii* or provide strain-specific identification. Counts are therefore reported as culturable populations assigned to the inoculated bacterial groups, rather than as molecularly confirmed strain counts. MRS and M17 recovery was likewise treated as presumptive because these media are not species-specific, and colonies were not confirmed by molecular identification.

Contaminating microflora was assessed concurrently. Moulds and yeasts were enumerated on YGC medium following aerobic incubation at 25 °C for 5 days. The Enterobacteriaceae population was determined using VRBG medium. These plates were overlaid with the same medium and incubated anaerobically at 37 °C for 24 h. All commercial media were prepared according to the manufacturer’s instructions.

Colony counts were converted to CFU/mL of beverage after accounting for dilution. Duplicate analytical determinations for a sample were averaged on the CFU/mL scale before conversion to log CFU/mL. The two replicate samples from each production experiment were retained separately for statistical analysis. Bacterial results are presented as means ± SD of the transformed observations, rounded to 1 decimal place. Plate counts quantify recovery under the specified conditions and do not distinguish all causes of reduced culturability. The approximate inoculation level was recorded on a mass basis (CFU/g), whereas the storage enumeration results are retained on their original volume basis (CFU/mL); no conversion between these bases was applied.

### 2.5. pH During Refrigerated Storage

Beverage pH was measured on days 0, 7, 14, 21 and 28 using an Elmetron pH meter (Elmetron, Zabrze, Poland) with temperature compensation, as described in Section 2.3. Values were recorded to two decimal places. Direct pH monitoring was used to track changes in hydrogen-ion activity; neither titratable acidity nor individual organic acids were determined.

### 2.6. Instrumental Hardness and Adhesive Force

Texture measurements were performed immediately after cooling the beverages following fermentation, designated as day 0, and after 14 and 28 days of refrigerated storage. Hardness and adhesive force were determined using a CT3 10K texture analyser (Brookfield Engineering Laboratories Inc., Middleborough, MA, USA) equipped with a TA4/1000 cylindrical acrylic probe with a flat end, a diameter of 38.1 mm, and a length of 20 mm. Before measurement, each sample was equilibrated at 20 °C. The samples were analysed without prior mixing. For each measurement, the probe was positioned centrally above the sample and moved towards and into the sample at a speed of 2.0 mm/s. Contact with the sample surface was detected at a trigger force of 0.04 N. The probe was introduced into the sample to a depth of 12 mm and subsequently withdrawn at a speed of 4.5 mm/s. A single compression cycle was applied during each measurement [14,17].

Hardness was defined as the maximum positive force during probe penetration. Adhesive force was the absolute value of the maximum negative force during withdrawal. Data were processed with TexturePro CT software, version 1.4 Build 17 (Brookfield Engineering Laboratories Inc., Middleborough, MA, USA). Two replicate samples were prepared within each of the two independent experiments, and two instrumental measurements were made per sample. Results are reported in N to one decimal place. These penetration measurements do not provide viscosity, storage modulus or yield stress.

### 2.7. Centrifugal Syneresis

Serum release under centrifugation was measured on days 0, 14 and 28. The centrifugal approach was consistent with that used to assess water retention in fermented cereal matrices [13,14], with the following settings used here. A 40.0 g portion was placed in a pre-weighed Falcon tube and centrifuged at 3250× *g* for 20 min at 4 °C. The separated supernatant was decanted, and the tube with sediment was reweighed. Released serum mass was obtained from the mass difference before and after decanting.

Centrifugal syneresis was calculated as S = 100 × a/b, where S is serum release (%), a is released serum mass (g), and b is initial beverage mass (g). Values are reported to one decimal place. This procedure measures separation under an imposed centrifugal load. It does not measure spontaneous separation in the storage container, and the aqueous phase is referred to as serum rather than whey.

### 2.8. Carbohydrate Analysis by HPLC-RI

Carbohydrate content was determined by high-performance liquid chromatography with refractive index detection (HPLC-RI) [14]. Samples were collected immediately after fermentation and after 14 and 28 days of refrigerated storage. For sample preparation, 8.00 g of each beverage was weighed into a Falcon-type centrifuge tube and mixed with 32.00 g of methanol. The samples were placed in an ultrasonic water bath at 30 °C for 30 min and subsequently centrifuged using an MPW-350R refrigerated laboratory centrifuge (MPW Med. Instruments, Warsaw, Poland) at 16,000× *g* for 30 min at 4 °C. After centrifugation, the clear supernatant was collected and filtered directly into chromatographic vials through 0.45 µm syringe filters (Merck, Darmstadt, Germany).

The HPLC system comprised a DeltaChrom pump, S 6020 injection valve, S 3580 refractive index detector and DeltaChrom column temperature controller (Sykam, Fürstenfeldbruck, Germany). The injection volume was 10 µL. Separation was performed using a COSMOSIL Sugar-D analytical column (4.6 mm internal diameter × 250 mm; Nacalai Tesque, Kyoto, Japan) and a guard column (4.6 mm × 10 mm; Nacalai Tesque, Kyoto, Japan). The mobile phase was acetonitrile (POCH, Gliwice, Poland) and deionised water, 60:40 (*w*/*w*), delivered isocratically at 1.0 mL/min. Column temperature was 30 °C. The refractive index detector used a signal range of 10,000 mV and an acquisition rate of 2 Hz.

Fructose, glucose, sucrose and raffinose were identified by comparison with authentic standards (Sigma-Aldrich, Burlington, MA, USA). Quantification used the respective peak areas and calibration relationships, accounting for the fivefold sample dilution (8.00 g of beverage in 40.00 g of extraction mixture). The reported extract calibration range was 0.01 to 2.00 g/100 mL, corresponding to a beverage working range of 0.50 to 100.00 mg/g in the analytical calculation. Calibration coefficients of determination exceeded 0.999. Table 1 reports the characteristics of the listed standard solutions and the signal-based quantitation thresholds; the standard concentrations shown are not the complete calibration series.

Signal-based limits of quantitation (LOQs) were determined at a signal-to-noise ratio (S/N) of 10 and corrected for sample dilution. The resulting beverage LOQs were 0.10 mg/g for fructose and glucose and 0.20 mg/g for sucrose and raffinose. These thresholds describe signal magnitude and must be distinguished from the lowest validated calibration level of 0.50 mg/g. Numerical concentrations below 0.50 mg/g were obtained by extrapolation and are reported only as estimated, semiquantitative values, including those above the signal-based LOQ. Fructose signals at estimated concentrations of 0.2 to 0.3 mg/g had S/N > 10, but their magnitude did not establish validated linear quantification at those concentrations. Numerical estimates below the carbohydrate-specific LOQ are additionally identified as below LOQ and are not interpreted as reliable quantitative measurements. Glucose below its LOQ is reported as <LOQ, not zero. No separate numerical limit of detection is reported. All beverage concentrations are expressed in mg/g.

### 2.9. Statistical Analysis

Statistical analyses were performed using Statgraphics Centurion XVII (Statgraphics Technologies, Inc., The Plains, VA, USA). For storage responses, the three fixed factors were storage time (A), flour addition (B; 0, 1, 2 and 3% *w*/*v*) and starter treatment (C). The factorial model included A, B, C, A × B, A × C, B × C and A × B × C, with a residual error term. Storage pH was measured on days 0, 7, 14, 21, and 28; bacterial populations, physical properties, and carbohydrates were assessed on days 0, 14, and 28. Each bacterial group was analysed separately using the two starter treatments that contained it. The other storage responses involved all three starter treatments. Fermentation profiles describe incubation time, which is distinct from storage time; the storage ANOVA probabilities are not assigned to fermentation kinetics.

The two replicate samples in each of the two independent production experiments were entered separately, giving four sample observations per combination. Duplicates were not averaged within a production experiment before ANOVA. The reported three-factor model does not include a separate production-batch term or an adjustment for dependence among samples sharing a batch. Its probabilities and Tukey groupings are treated as nominal, exploratory results. Independent production replication remained *n* = 2. Opening separate jars at each time point avoided repeated sampling of a single jar but did not eliminate the shared-batch structure.

Residual normality and homogeneity of variance were checked using the Shapiro–Wilk and Levene tests, respectively. Bacterial observations expressed as log CFU/mL met the stated requirements for parametric testing. Tukey’s honest significant difference (HSD) procedure used a nominal α of 0.05 for comparisons between experimental combinations. Passing these assumption checks does not establish independence of observations. The bacterial tables contain 24 combinations, whereas the physical property tables contain 36. Means and standard deviations are reported.

Table 8 presents the available nominal probabilities for storage pH, hardness, centrifugal syneresis and the three bacterial groups. No factorial probabilities are assigned to adhesive force because the separate statistical summary labelled adhesiveness in g·s refers to a different dimensional quantity from the maximum withdrawal force reported here in *N. Carbohydrate* trends, particularly extrapolated concentrations, are interpreted descriptively. The original figure groupings are retained as nominal annotations and are not used to establish metabolic mechanisms or confirm differences between independent production batches.

## 3. Results

### 3.1. Acidification Profiles

**Figure 1:**
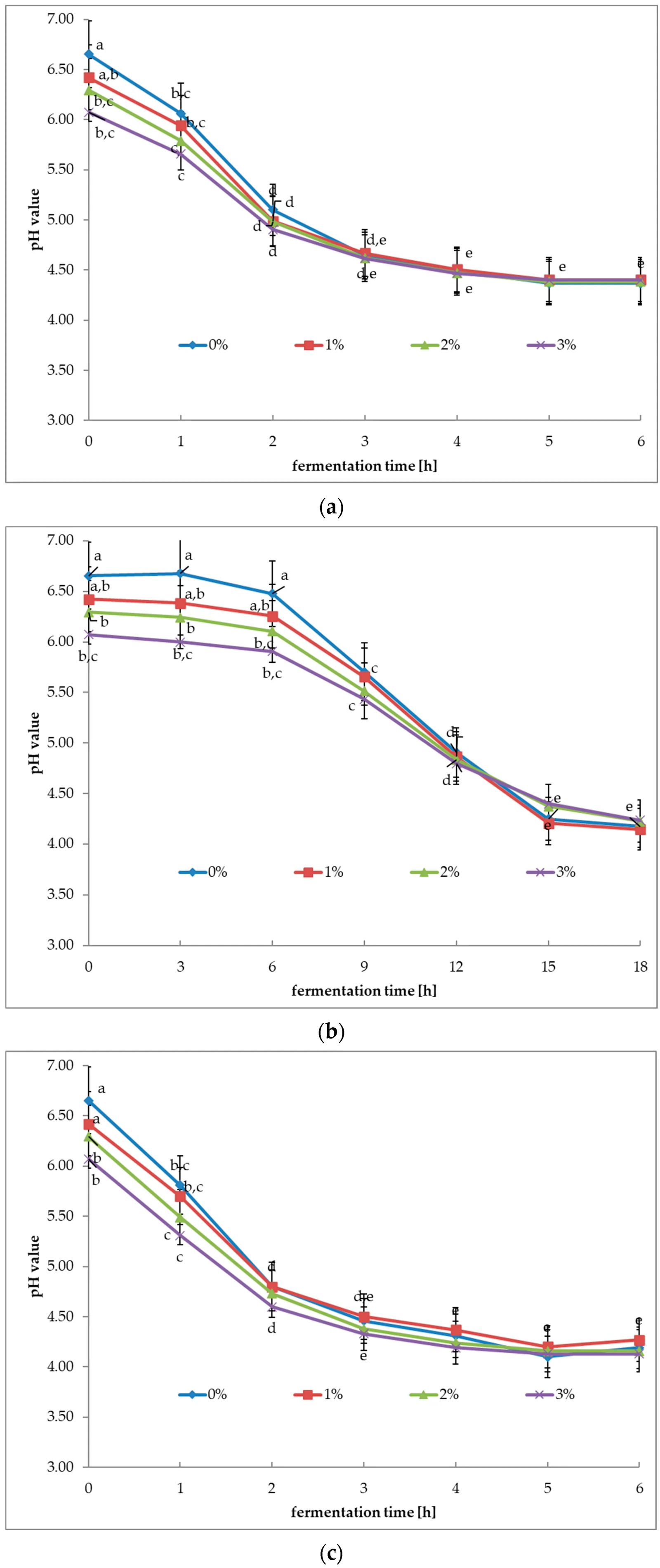
Acidification profiles at 37 °C for beverages fermented with (**a**) yoghurt starter, (**b**) propionic starter and (**c**) both starters. Incubation lasted 6 h in panels a and c and 18 h in panel (**b**). Legends indicate added millet flour (% *w*/*v*) (means and SD values; letters indicate homogeneous groups at *p*-value = 0.05).

The initial pH was highest in the beverage without added flour and decreased with increasing flour supplementation (Figure 1). In treatments containing yoghurt bacteria, most of the decline in pH occurred during the first 3 h. At the 6 h endpoint, the yoghurt treatment was approximately pH 4.4, and the mixed treatment was approximately pH 4.1 to 4.2. The propionic starter alone acidified more slowly, with the main decrease between approximately 6 and 15 h and a final pH near 4.2 at 18 h. Flour-dependent differences were smaller at the end of fermentation than at the time of inoculation. These profiles compare the treatments at 37 °C and do not establish their relative performance at individually optimised temperatures.

### 3.2. Culturable Bacteria Populations

Post-fermentation presumptive lactobacilli counts were 7.3 to 7.4 log CFU/mL (Table 2). Counts remained close to these values through day 14. By day 28, yoghurt-only beverages contained 7.8 to 8.2 log CFU/mL, whereas mixed-culture beverages contained 7.2 to 7.7 log CFU/mL. Thus, the late increase seen in yoghurt-only treatments was less evident in the mixed treatments.

Post-fermentation presumptive *S. thermophilus* counts were 8.5 to 8.6 log CFU/mL (Table 3). Changes during the first 14 days depended on formulation. Counts at day 28 were lower than immediately after fermentation in all treatments. The lowest value, 7.0 log CFU/mL, occurred in the mixed-culture beverage containing 2% flour, not in a propionic-only treatment.

Presumptive *Propionibacterium* spp. counts were 7.1 log CFU/mL after fermentation with the propionic starter alone and 7.9 log CFU/mL with both starters (Table 4). All day-28 counts were lower than their respective day-0 values. The trajectories were not uniformly monotonic: mixed treatments with 2% and 3% flour showed higher counts on day 28 than on day 14. At day 28, mixed treatments contained 6.1 to 6.7 log CFU/mL, compared with 5.2 to 5.7 log CFU/mL for propionic-only treatments. The highest final value occurred with 2% flour and both starters.

Higher final propionibacteria counts in mixed treatments did not imply uniformly smaller losses during storage. Calculated from the reported means, the day-0 to day-28 decrease with 2% flour was 1.2 log units in mixed culture and 1.9 log units with propionibacteria alone. Without flour, the corresponding decreases were 1.8 and 1.7 log units. These descriptive differences distinguish the post-fermentation population level from subsequent retention; they are not additional statistical tests of change scores.

### 3.3. pH During Refrigerated Storage

**Figure 2:**
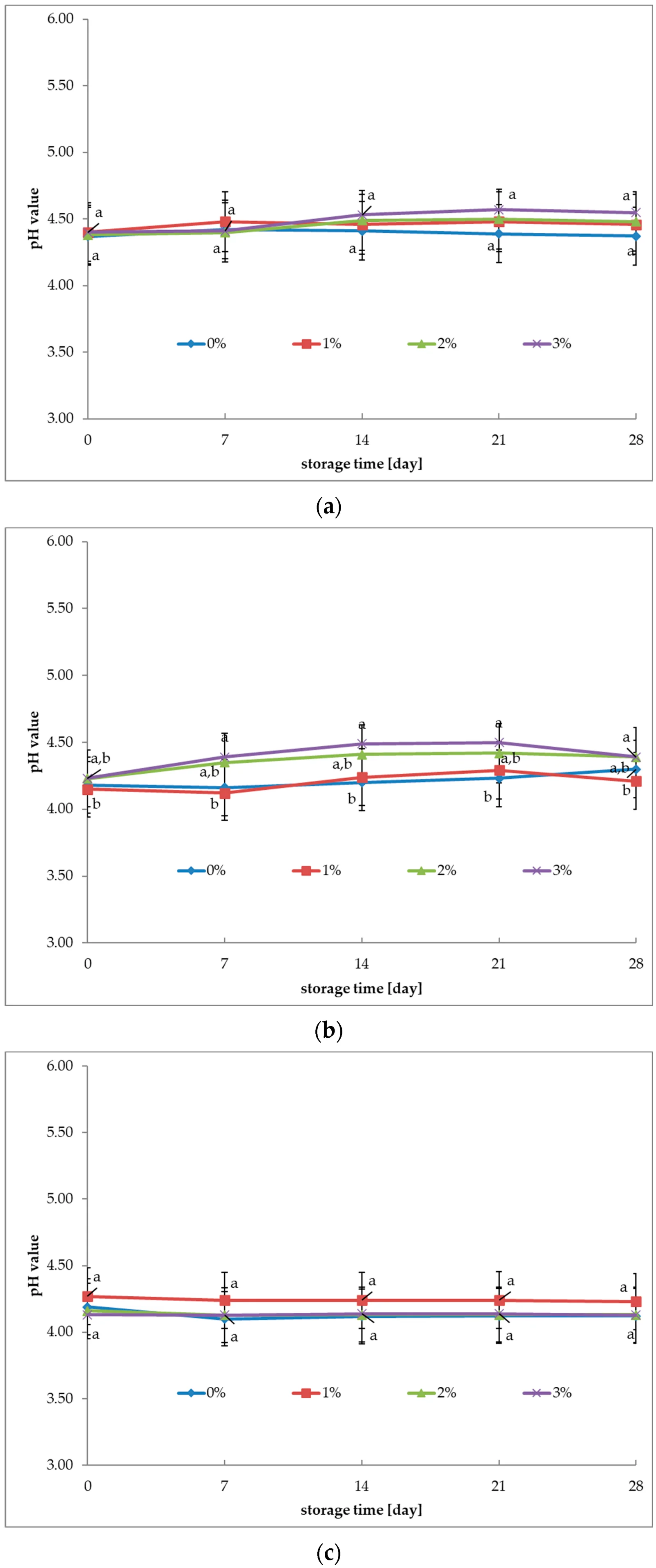
pH during storage at 6 °C after fermentation with (**a**) yoghurt starter, (**b**) propionic starter and (**c**) both starters. Legends indicate added millet flour (% *w*/*v*) (means and SD values; letters indicate homogeneous groups at *p*-value = 0.05).

Yoghurt-only beverages showed limited pH changes during storage, with the 3% flour formulation approaching pH 4.5 (Figure 2a). Propionic-only beverages showed somewhat larger fluctuations, particularly at 2% and 3% flour (Figure 2b). Mixed-culture beverages remained at approximately pH 4.1–4.2 throughout the storage period (Figure 2c). These observations show pH stability under the tested conditions. They do not identify the acids present or establish that one culture inhibited acid formation by another.

### 3.4. Hardness, Adhesive Force and Centrifugal Syneresis

Hardness depended on both formulation and starter treatment (Table 5). Beverages without flour had a hardness of 0.2 N throughout storage. With the propionic starter alone, hardness remained between 0.2 and 0.5 N. In yoghurt and mixed treatments, higher flour addition was associated with greater hardness, reaching 1.6 and 1.7 N, respectively, at 3% flour on day 0. The day-28 mixed treatment with 1% flour had a hardness of 0.4 ± 0.1 N. Similar final pH values across the starter treatments did not produce equivalent penetration forces.

Adhesive force ranged from 0.0 to 0.2 N (Table 6). Values of 0.2 N occurred principally in yoghurt-containing treatments with higher flour additions during storage. These forces were small and are reported as instrumental responses without assigning a sensory acceptability threshold.

Centrifugal serum release was high in formulations without flour, reaching 94.1% to 95.2% on day 28 (Table 7). Flour generally reduced release, but the response differed among starter treatments. With 3% flour, day-28 release was 48.3% with the yoghurt starter, 68.9% with the propionic starter and 57.8% with both starters. The mixed treatment, therefore, did not match the lowest syneresis of the yoghurt-only treatment. Moreover, its 3% flour value increased slightly from 56.0% on day 0 to 57.8% on day 28, whereas the yoghurt-only value decreased from 56.7% to 48.3%.

The lowest serum release and the highest final propionibacteria count occurred in different formulations. The physical measurements, therefore, indicate a formulation trade-off rather than a single optimum for all responses.

### 3.5. Residual Carbohydrates During Storage

Glucose was below its signal-based LOQ of 0.10 mg/g. Estimated fructose concentrations were 0.2–0.3 mg/g, with little apparent variation. Although fructose signals had S/N > 10, these values were extrapolated below the lowest validated calibration level of 0.50 mg/g and are semiquantitative. They cannot support precise estimates of fructose consumption. The assay focused on selected residual sugars, not on total carbohydrate content or a fermentation carbon balance.

**Figure 3:**
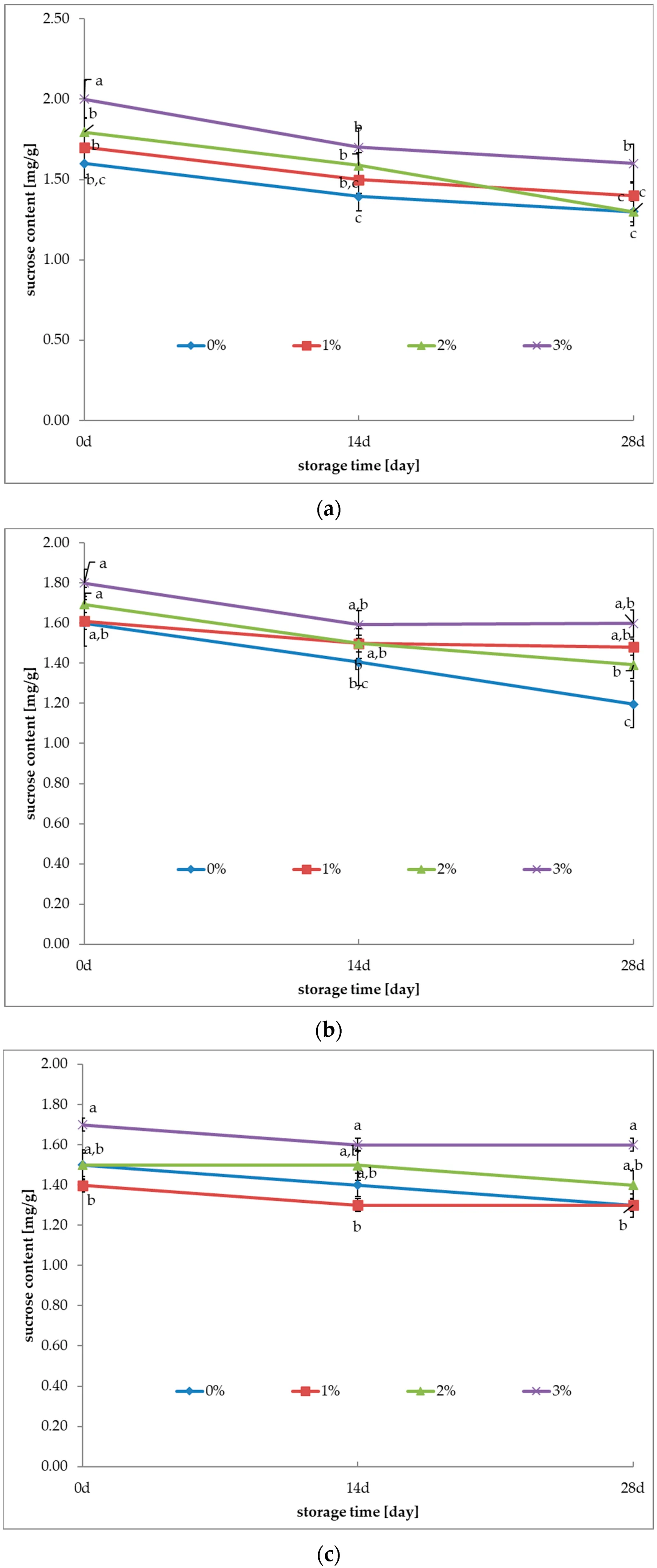
Sucrose during storage at 6 °C after fermentation with (**a**) yoghurt starter, (**b**) propionic starter and (**c**) both starters. Concentrations are in mg/g of beverage. Legends indicate added flour (% *w*/*v*). Concentrations below 0.50 mg/g, if present, are extrapolated estimates rather than validated quantitative results (means and SD values; letters indicate homogeneous groups at *p*-value = 0.05).

Sucrose generally decreased during refrigerated storage (Figure 3). The direction and magnitude of the changes depended on starter treatment and flour concentration, while some trajectories remained nearly unchanged for part of the storage period. Such changes are compatible with continued carbohydrate conversion but cannot identify the organism responsible or establish a specific metabolic pathway.

**Figure 4:**
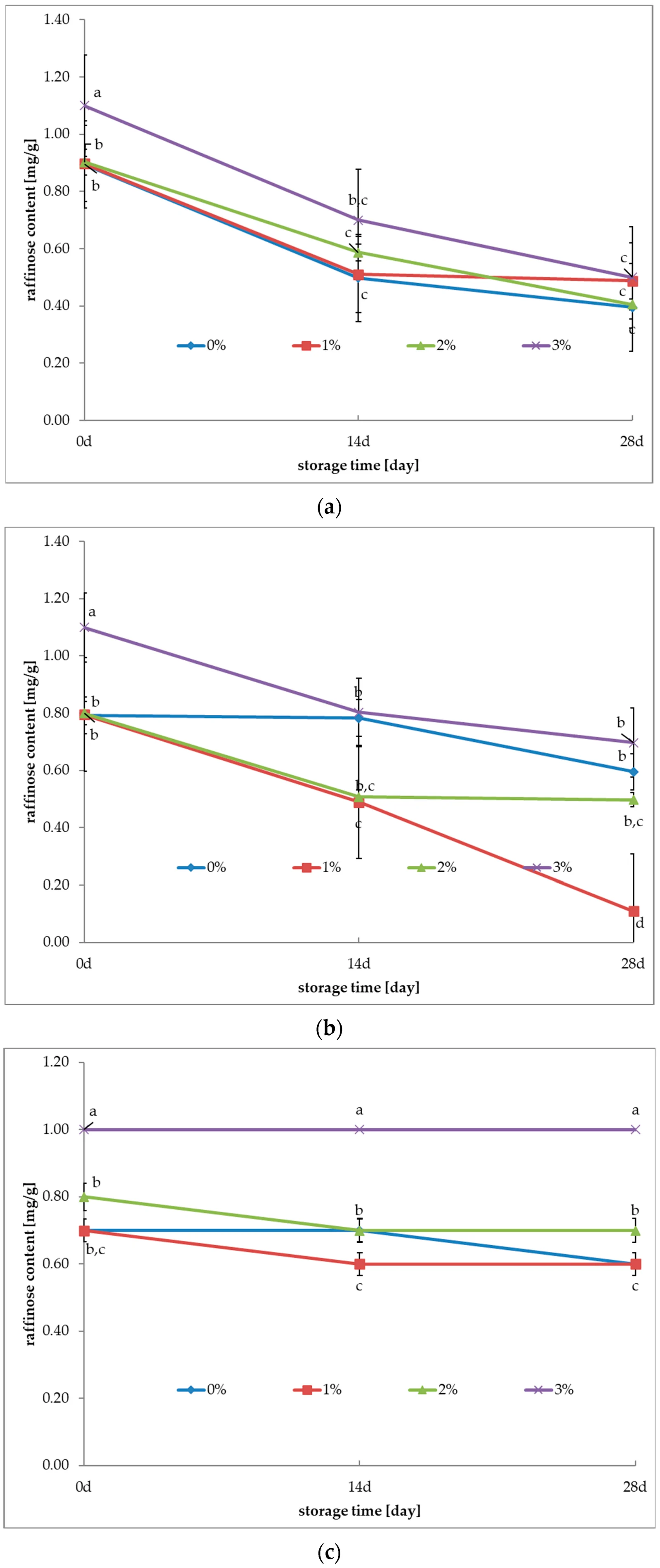
Raffinose during storage at 6 °C after fermentation with (**a**) yoghurt starter, (**b**) propionic starter and (**c**) both starters. Concentrations are in mg/g of beverage. Legends indicate added flour (% *w*/*v*). Every concentration below 0.50 mg/g is an estimated, semiquantitative result from calibration extrapolation. The approximately 0.1 mg/g point in panel b (1% flour, day 28) is additionally below the LOQ of 0.20 mg/g. Letters reproduce nominal comparisons and do not validate extrapolated concentrations or establish independent batch effects (means and SD values; letters indicate homogeneous groups at *p*-value = 0.05).

Raffinose decreased in several formulations, with larger apparent changes in yoghurt-only and some propionic-only treatments (Figure 4). Mixed treatments showed smaller changes, and the 3% flour treatment was approximately stable. All plotted concentrations below 0.50 mg/g are semiquantitative estimates from calibration extrapolation. In particular, the approximately 0.1 mg/g value for the propionic starter with 1% flour on day 28 is also below the raffinose LOQ of 0.20 mg/g. Its numerical value is retained as an estimate and cannot establish the magnitude of depletion. These observations do not demonstrate mutualism, cross-feeding or α-galactosidase activity.

### 3.6. Exploratory Factorial Analysis

The reported three-factor model yielded nominal storage time × flour × starter interactions for presumptive lactobacilli (*p* = 0.011), presumptive *Propionibacterium* spp. (*p* = 0.006) and centrifugal syneresis (*p* = 0.021). The corresponding probabilities were 0.284 for presumptive *S. thermophilus*, 0.312 for pH and 0.612 for hardness (Table 8). These outcomes suggest response-specific patterns but are exploratory because adjustment for samples sharing a production batch is not documented. A statistical interaction does not establish metabolic synergy.

## 4. Discussion

The study identified two partly separate responses to formulation. Starter composition was associated with acidification profiles and recoverable bacterial populations, whereas the addition of flour had a pronounced effect on penetration force and centrifugal serum release. The mixed starter achieved rapid acidification and higher final propionibacteria counts, but the yoghurt-only treatment with 3% flour had the lowest centrifugal syneresis. These outcomes support a technological comparison of starter systems, not a general claim that co-fermentation improves every property.

The rapid decrease in pH with yoghurt-containing cultures is consistent with their established use as acidifying starters. Metabolite exchange between *S. thermophilus* and *L. delbrueckii* subsp. *bulgaricus* has been demonstrated in milk [7,8]. Nevertheless, the present experiment did not include the two yoghurt species separately, so it cannot demonstrate that the same protocooperation occurred in millet. Fermented millet and oat studies likewise show that culture selection affects acidification and product characteristics [4,5,6]. These comparisons support the feasibility of cereal fermentation while leaving the mechanism in this commercial starter unresolved.

Incubation at 37 °C is an important boundary of the comparison. It exceeded the manufacturer’s application temperature for the propionic starter and was slightly below the recommended yoghurt range. The longer acidification period with propionibacteria alone could reflect temperature, substrate availability, adaptation and culture physiology. Thermal stress cannot be assigned as the cause because no temperature series or direct stress measurements were included. Similarly, the 18 h propionic treatment and 6 h yoghurt-containing treatments were not sampled after equal incubation periods. Their post-fermentation populations should therefore be interpreted as outcomes of the tested processes, rather than as intrinsic growth-rate comparisons.

The absence of yoghurt-starter growth in the sodium lactate agar control addresses a specific potential source of background colonies in mixed samples. Its relevance is confined to the tested starter and incubation conditions. Sodium lactate agar should not be described as universally species-selective, and the counts do not provide molecular confirmation of strain identity. The use of the same recovery temperature across treatments also does not establish maximal recovery of all viable cells. Strain-specific enumeration would strengthen subsequent work. Strain identifiers were unavailable for the commercial preparations; species composition was based on supplier declarations. Consequently, the results cannot be generalised to all strains of the named species.

The higher propionibacteria counts in mixed treatments are consistent with a supportive association. Tarnaud et al. [10] showed that the response of *P. freudenreichii* to soy and dairy environments depends on adaptation and its microbial context. Tangyu et al. [11] demonstrated cooperative upgrading of sunflower seed beverage with *P. freudenreichii* and *Bacillus amyloliquefaciens*, whereas Gungor et al. [12] examined propionibacteria in other plant beverages. These studies establish biological plausibility but involve different substrates or partners. In the present study, lactate provision, peptide exchange and changes in redox conditions are hypotheses, because the relevant substrates and metabolites were not quantified.

Inoculum composition adds another limitation. Each starter was dosed at 0.04% in mixed treatments, so the total commercial preparation added was twice that used in single starter treatments. Initial beverage counts were approx. 7 log CFU/g, including in the mixed treatment, but this approximate level does not demonstrate equal initial populations of each bacterial group. Commercial preparations may also contribute carrier material. The experiment, therefore, cannot separate all effects of microbial association, inoculum composition and formulation. Moreover, a higher final count is not synonymous with improved fractional survival: without flour, the mixed treatment had a slightly greater log decline despite its higher day 28 population. The strongest descriptive retention advantage was observed with 2% flour under the specific conditions tested.

The smaller late increase in lactobacilli in mixed beverages may reflect nutrient competition or changes in the chemical environment. Different stress responses during cold storage are well documented in fermented milk [18,19]. However, assigning the current pattern to propionate or acetate inhibition would require measuring them. Stable pH does not establish the absence of acid production, because pH also depends on buffering and acid–base equilibria. Neither the acid balance nor the contribution of proteolysis can be resolved from viable counts and pH alone.

Niamah et al. [20] compared yoghurt starters supplemented with different probiotic bacteria or yeast and measured both storage viability and volatile compounds. Their findings illustrate that starter combinations can alter populations and aroma, but the dairy substrate and adjunct organisms differ from those used here. The present study did not measure volatiles or sensory responses, so higher counts cannot be translated into improved flavour. Horlacher et al. [15] similarly related the potential nutritional benefits of traditional plant fermentation to process conditions and microbial activity. Those benefits provide a rationale for further work, not evidence that the millet beverages gained nutritional or health properties.

Flour supplied additional matrix material, which is consistent with increased penetration force and reduced centrifugal release. Heating can promote starch swelling and gelatinisation, while flour proteins, fibre and particles can contribute to water retention. These mechanisms were not distinguished experimentally. The proximate compositions were manufacturer declarations rather than analytical measurements of the actual batches, so changes in protein, starch or fibre cannot be quantitatively linked to the observed texture. The similar final pH among some treatments, despite different hardness levels, also argues against explaining the structure solely by protein coagulation at low pH.

Studies of fermented gels have demonstrated effects of solids content and EPS-producing cultures on water retention and mechanics [19,21,22]. EPS-associated changes have also been reported in a finger millet beverage [5]. Here, EPS-producing capacity was a supplier characteristic of the starter, not a measured outcome in millet. Consequently, neither EPS accumulation nor its contribution to hardness has been demonstrated. Penetration force measures resistance under a specified geometry and deformation rate; it cannot substitute for flow curves, elastic modulus, yield stress or microstructure.

Even the best formulations released substantial serum during centrifugation. This indicates limited resistance to the imposed load and prevents describing the matrix as physically stable without qualification. However, a release of 48.3% or 57.8% under 3250× *g* does not mean that the same fraction separates spontaneously in a refrigerated package. Comparisons with rice and oat beverages [13,14] also require matching centrifugation force, duration and temperature. No commercial acceptance threshold was established for this method and product. Spontaneous separation, dispersibility, viscosity, and sensory testing are required before judging suitability for use as a ready-to-drink beverage or yoghurt-like product.

The residual carbohydrate results provide only a partial view of substrate conversion. The assay covered four sugars and did not quantify the complete carbohydrate pool or hydrolysis products. Changes in sucrose and raffinose are consistent with continued microbial conversion at 6 °C, but the absence of an uninoculated storage control and a carbon balance limit causal inference. Glucose below the LOQ does not establish its absence or rapid consumption. Nor does a decline in raffinose identify the responsible strain or enzyme. Organic acid measurements at fermentation and storage sampling points would be needed to connect carbohydrate loss with product formation. Analytical uncertainty further limits interpretation at low concentrations. A signal exceeding the S/N threshold does not validate extrapolation below the calibration range. Fructose estimates of 0.2 to 0.3 mg/g and raffinose estimates below 0.50 mg/g therefore provide only semiquantitative information. Values below the carbohydrate-specific signal-based LOQ warrant still greater caution. Neither small differences between such estimates nor their apparent changes over time establish a quantitative metabolic response.

The objectives concerning observable technological responses were addressed, whereas metabolic mechanisms and commercial suitability remain unresolved. Only two independent production experiments were conducted. Entering duplicate samples separately creates a risk of pseudoreplication if shared batch dependence is not accounted for. No such adjustment is documented for the reported analysis, so uncertainty may be underestimated. The nominal interactions in Table 8, therefore, cannot replace an analysis respecting the batch structure. An interaction between storage time, flour, and starter treatment indicates a conditional pattern in the response, not biological synergy or mutualism. Further inference requires the original sample data and a model that accounts for production batch, or an analysis based on batch summaries, supported by additional independent production experiments. Subsequent work should also compare temperatures and inoculation sequences, include matched inoculum controls and strain-resolved counts, and measure lactate, propionate, acetate, free amino nitrogen, and EPS. Rheological, spontaneous separation and sensory measurements are needed to evaluate product suitability.

## 5. Conclusions

Under the tested fermentation and storage conditions, yoghurt containing starters acidified rapidly, and mixed cultures yielded higher absolute counts of presumptive propionibacteria. Millet flour increased penetration hardness and reduced centrifugal serum release, but the highest final bacterial count and the lowest serum release occurred in different formulations. These observations support further investigation of fermented millet beverages without establishing metabolic synergy, mutualism or probiotic efficacy. Interpretation is limited by two independent production experiments, an analysis without a documented adjustment for shared batch structure, and semiquantitative carbohydrate estimates that fall below the validated calibration range. Direct metabolite measurements and rheological, storage separation and sensory assessments remain necessary before mechanisms or commercial suitability can be established.

## Figures and Tables

**Table 1:** Carbohydrate standards used for HPLC-RI analysis.

Carbohydrate	Retention Time (min)	Peak Area (mV × s)	Standard (g/100 mL)	LOQ (mg/g Beverage)	Lowest Validated Level (mg/g Beverage)
Fructose	7.717	1846	0.04	0.10	0.50
Glucose	7.880	1619	0.04	0.10	0.50
Sucrose	9.600	971	0.40	0.20	0.50
Raffinose	12.208	361	0.40	0.20	0.50

LOQ is the signal-based threshold at S/N = 10 after dilution correction. Every beverage result below 0.50 mg/g is estimated by extrapolation and is semiquantitative, even if above this LOQ. Values below the respective LOQ have additional uncertainty. The listed standard solutions do not constitute the full calibration series.

**Table 2:** Presumptive lactobacilli during storage at 6 °C in beverages fermented with yoghurt starter alone or yoghurt and propionic starters together (log CFU/mL).

Storage Time	Addition of Millet Flour	Population in Samples of Millet Beverages (With Various Additions of Millet Flour) Fermented by:	
		Yoghurt Starter Culture	Yoghurt and Propionic Starter Cultures
0 d	0%	7.3 ± 0.1 ^a^	7.4 ± 0.2 ^a,b^
	1%	7.3 ± 0.2 ^a^	7.4 ± 0.1 ^a,b^
	2%	7.3 ± 0.1 ^a^	7.4 ± 0.1 ^a,b^
	3%	7.3 ± 0.1 ^a^	7.4 ± 0.2 ^a,b^
14 d	0%	7.3 ± 0.2 ^a^	7.5 ± 0.1 ^a,b^
	1%	7.2 ± 0.2 ^a^	7.6 ± 0.1 ^a,b^
	2%	7.5 ± 0.1 ^a,b^	7.7 ± 0.2 ^b,c^
	3%	7.5 ± 0.1 ^a,b^	7.5 ± 0.2 ^a,b^
28 d	0%	8.2 ± 0.1 ^d^	7.3 ± 0.2 ^a^
	1%	7.8 ± 0.1 ^b,c,d^	7.4 ± 0.2 ^a,b^
	2%	8.0 ± 0.1 ^c,d^	7.7 ± 0.1 ^b,c^
	3%	7.8 ± 0.2 ^b,c,d^	7.2 ± 0.1 ^a^

^a–d^ Superscript lowercase letters within the entire table indicate statistically significant differences (*p* < 0.05) among all experimental combinations (interaction effect: storage time × flour addition × culture type) according to a three-way analysis of variance followed by Tukey’s honest significant difference (HSD) post hoc test.

**Table 3:** Presumptive *S. thermophilus* during storage at 6 °C in beverages fermented with yoghurt starter alone or yoghurt and propionic starters together (log CFU/mL).

Storage Time	Addition of Millet Flour	Population in Samples of Millet Beverages (With Various Additions of Millet Flour) Fermented by:	
		Yoghurt Starter Culture	Yoghurt and Propionic Starter Cultures
0 d	0%	8.6 ± 0.1 ^b,c^	8.5 ± 0.1 ^c,d^
	1%	8.6 ± 0.1 ^b,c^	8.5 ± 0.1 ^c,d^
	2%	8.6 ± 0.2 ^b,c^	8.5 ± 0.1 ^c,d^
	3%	8.6 ± 0.2 ^b,c^	8.5 ± 0.2 ^c,d^
14 d	0%	8.8 ± 0.1 ^a^	8.4 ± 0.2 ^d,e^
	1%	8.1 ± 0.1 ^g^	8.2 ± 0.1 ^f,g^
	2%	8.7 ± 0.1 ^a,b^	8.1 ± 0.1 ^g^
	3%	8.4 ± 0.2 ^d,e^	8.3 ± 0.2 ^e,f^
28 d	0%	8.1 ± 0.1 ^g^	7.9 ± 0.1 ^h^
	1%	7.8 ± 0.2 ^h^	7.8 ± 0.2 ^h^
	2%	7.9 ± 0.1 ^h^	7.0 ± 0.1 ^i^
	3%	7.9 ± 0.1 ^h^	7.9 ± 0.2 ^h^

^a–i^ Superscript lowercase letters within the entire table indicate statistically significant differences (*p* < 0.05) among all experimental combinations (interaction effect: storage time × flour addition × culture type) according to a three-way analysis of variance followed by Tukey’s honest significant difference (HSD) post hoc test.

**Table 4:** Presumptive *Propionibacterium* spp. during storage at 6 °C in beverages fermented with propionic starter alone or yoghurt and propionic starters together (log CFU/mL).

Storage Time	Addition of Millet Flour	Population in Samples of Millet Beverages (With Various Additions of Millet Flour) Fermented by:	
		Propionic Starter Culture	Yoghurt and Propionic Starter Cultures
0 d	0%	7.1 ± 0.1 ^b^	7.9 ± 0.1 ^a^
	1%	7.1 ± 0.1 ^b^	7.9 ± 0.1 ^a^
	2%	7.1 ± 0.1 ^b^	7.9 ± 0.1 ^a^
	3%	7.1 ± 0.1 ^b^	7.9 ± 0.2 ^a^
14 d	0%	6.2 ± 0.2 ^e^	6.9 ± 0.2 ^c^
	1%	6.2 ± 0.2 ^e^	6.8 ± 0.1 ^c^
	2%	5.8 ± 0.1 ^f^	6.4 ± 0.2 ^d^
	3%	5.6 ± 0.2 ^g^	6.3 ± 0.2 ^d^
28 d	0%	5.4 ± 0.1 ^g^	6.1 ± 0.2 ^e^
	1%	5.5 ± 0.2 ^g^	6.1 ± 0.1 ^e^
	2%	5.2 ± 0.2 ^h^	6.7 ± 0.1 ^c^
	3%	5.7 ± 0.2 ^f^	6.4 ± 0.1 ^d^

^a–h^ Superscript lowercase letters within the entire table indicate statistically significant differences (*p* < 0.05) among all experimental combinations (interaction effect: storage time × flour addition × culture type) according to a three-way analysis of variance followed by Tukey’s honest significant difference (HSD) post hoc test.

**Table 5:** Instrumental hardness during storage at 6 °C in beverages fermented with yoghurt starter, propionic starter or both starters together (N).

Storage Time	Addition of Millet Flour	Samples of Millet Beverages (With Various Additions of Millet Flour) Fermented by:		
		Yoghurt Starter Culture	Propionic Starter Culture	Yoghurt and Propionic Starter Cultures
0 d	0%	0.2 ± 0.1 ^a^	0.2 ± 0.1 ^a^	0.2 ± 0.1 ^a^
	1%	0.3 ± 0.1 ^a^	0.3 ± 0.1 ^a^	0.3 ± 0.1 ^a^
	2%	0.7 ± 0.3 ^c^	0.3 ± 0.1 ^a^	0.8 ± 0.2 ^c^
	3%	1.6 ± 0.1 ^e^	0.4 ± 0.1 ^a,b^	1.7 ± 0.2 ^e^
14 d	0%	0.2 ± 0.1 ^a^	0.2 ± 0.0 ^a^	0.2 ± 0.1 ^a^
	1%	0.4 ± 0.1 ^a,b^	0.4 ± 0.1 ^a,b^	0.4 ± 0.2 ^a,b^
	2%	1.0 ± 0.2 ^d^	0.5 ± 0.2 ^b^	0.9 ± 0.1 ^d^
	3%	1.4 ± 0.2 ^e^	0.4 ± 0.1 ^a,b^	1.4 ± 0.1 ^e^
28 d	0%	0.2 ± 0.0 ^a^	0.2 ± 0.0 ^a^	0.2 ± 0.0 ^a^
	1%	0.3 ± 0.1 ^a^	0.2 ± 0.0 ^a^	0.4 ± 0.1 ^a,b^
	2%	1.0 ± 0.2 ^d^	0.3 ± 0.1 ^a^	0.9 ± 0.2 ^d^
	3%	1.4 ± 0.2 ^e^	0.4 ± 0.1 ^a,b^	1.4 ± 0.2 ^e^

^a–e^ Superscript lowercase letters within the entire table indicate statistically significant differences (*p* < 0.05) among all experimental combinations (interaction effect: storage time × flour addition × culture type) according to a three-way analysis of variance followed by Tukey’s honest significant difference (HSD) post hoc test.

**Table 6:** Maximum adhesive force during probe withdrawal, measured during storage at 6 °C in beverages fermented with yoghurt starter, propionic starter or both starters together (N).

Storage Time	Addition of Millet Flour	Samples of Millet Beverages (With Various Additions of Millet Flour) Fermented by:		
		Yoghurt Starter Culture	Propionic Starter Culture	Yoghurt and Propionic Starter Cultures
0 d	0%	0.0 ± 0.0 ^a^	0.0 ± 0.0 ^a^	0.0 ± 0.0 ^a^
	1%	0.1 ± 0.0 ^b^	0.0 ± 0.0 ^a^	0.1 ± 0.0 ^b^
	2%	0.1 ± 0.0 ^b^	0.1 ± 0.1 ^b^	0.1 ± 0.0 ^b^
	3%	0.1 ± 0.0 ^b^	0.1 ± 0.0 ^b^	0.1 ± 0.0 ^b^
14 d	0%	0.0 ± 0.0 ^a^	0.0 ± 0.0 ^a^	0.0 ± 0.0 ^a^
	1%	0.1 ± 0.0 ^b^	0.0 ± 0.0 ^a^	0.1 ± 0.0 ^b^
	2%	0.1 ± 0.0 ^b^	0.1 ± 0.1 ^b^	0.1 ± 0.0 ^b^
	3%	0.2 ± 0.1 ^c,d^	0.1 ± 0.0 ^b^	0.2 ± 0.1 ^c,d^
28 d	0%	0.0 ± 0.0 ^a^	0.1 ± 0.0 ^b^	0.0 ± 0.0 ^a^
	1%	0.1 ± 0.0 ^b^	0.0 ± 0.0 ^a^	0.1 ± 0.0 ^b^
	2%	0.2 ± 0.1 ^c,d^	0.1 ± 0.1 ^b^	0.2 ± 0.1 ^c,d^
	3%	0.2 ± 0.1 ^c,d^	0.1 ± 0.1 ^b^	0.2 ± 0.1 ^c,d^

^a–d^ Superscript lowercase letters within the entire table indicate statistically significant differences (*p* < 0.05) among all experimental combinations (interaction effect: storage time × flour addition × culture type) according to a three-way analysis of variance followed by Tukey’s honest significant difference (HSD) post-hoc test.

**Table 7:** Centrifugal syneresis during storage at 6 °C in beverages fermented with yoghurt starter, propionic starter or both starters together (% released serum by mass).

Storage Time	Addition of Millet Flour	Samples of Millet Beverages (With Various Additions of Millet Flour) Fermented by:		
		Yoghurt Starter Culture	Propionic Starter Culture	Yoghurt and Propionic Starter Cultures
0 d	0%	90.0 ± 0.2 ^i^	94.9 ± 0.1 ^i^	89.0 ± 0.1 ^i^
	1%	76.0 ± 0.2 ^g^	82.0 ± 0.2 ^h^	75.4 ± 0.2 ^g^
	2%	60.8 ± 0.3 ^d^	62.4 ± 0.2 ^e^	61.2 ± 0.3 ^d^
	3%	56.7 ± 0.1 ^c^	67.6 ± 0.3 ^f^	56.0 ± 0.3 ^c^
14 d	0%	92.5 ± 0.2 ^i^	94.8 ± 0.3 ^i^	91.4 ± 0.2 ^i^
	1%	76.0 ± 0.1 ^g^	81.2 ± 0.1 ^h^	75.8 ± 0.1 ^g^
	2%	58.9 ± 0.3 ^d^	62.8 ± 0.2 ^e^	60.5 ± 0.2 ^d^
	3%	52.5 ± 0.2 ^b^	63.2 ± 0.2 ^e^	56.4 ± 0.2 ^c^
28 d	0%	95.2 ± 0.1 ^i^	94.7 ± 0.1 ^i^	94.1 ± 0.1 ^i^
	1%	76.0 ± 0.2 ^g^	80.5 ± 0.1 ^h^	77.0 ± 0.1 ^g^
	2%	57.1 ± 0.2 ^c^	63.3 ± 0.3 ^e^	66.4 ± 0.2 ^f^
	3%	48.3 ± 0.1 ^a^	68.9 ± 0.2 ^f^	57.8 ± 0.3 ^c^

^a–i^ Superscript lowercase letters within the entire table indicate statistically significant differences (*p* < 0.05) among all experimental combinations (interaction effect: storage time × flour addition × culture type) according to a three-way analysis of variance followed by Tukey’s honest significant difference (HSD) post-hoc test.

**Table 8:** Nominal probabilities from the reported three factor ANOVA for storage responses.

Carbohydrate	A	B	C	A × B	A × C	B × C	A × B × C
pH	<0.001	<0.001	0.002	0.015	0.041	0.182	0.312
Centrifugal syneresis	<0.001	<0.001	0.042	0.001	0.215	0.089	0.021
Hardness	0.008	<0.001	0.154	0.031	0.380	0.421	0.612
Adhesive force	<0.001	<0.001	0.018	0.006	0.142	0.025	0.041
Presumptive lactobacilli	<0.001	0.002	<0.001	0.014	0.008	0.032	0.011
Presumptive *S. thermophilus*	<0.001	0.041	<0.001	0.112	0.019	0.205	0.284
Presumptive *Propionibacterium* spp.	<0.001	<0.001	<0.001	0.003	0.025	0.012	0.006

A, storage time; B, flour addition; C, starter treatment. Values are *p*-values, with nominal α = 0.05. Four sample observations per combination came from two independent production experiments.

## Data Availability

The authors confirm that the data supporting the findings of this study are available within the article.
